# Assessing the Response of Nematode Communities to Climate Change-Driven Warming: A Microcosm Experiment

**DOI:** 10.1371/journal.pone.0066653

**Published:** 2013-06-18

**Authors:** Ruth Gingold, Tom Moens, Axayácatl Rocha-Olivares

**Affiliations:** 1 Biological Oceanography Department, Centro de Investigación Científica y de Educación Superior de Ensenada, Ensenada, Baja California, Mexico; 2 Department of Biology, Ghent University, Ghent, Flanders, Belgium; National Institute of Water & Atmospheric Research, New Zealand

## Abstract

Biodiversity has diminished over the past decades with climate change being among the main responsible factors. One consequence of climate change is the increase in sea surface temperature, which, together with long exposure periods in intertidal areas, may exceed the tolerance level of benthic organisms. Benthic communities may suffer structural changes due to the loss of species or functional groups, putting ecological services at risk. In sandy beaches, free-living marine nematodes usually are the most abundant and diverse group of intertidal meiofauna, playing an important role in the benthic food web. While apparently many functionally similar nematode species co-exist temporally and spatially, experimental results on selected bacterivore species suggest no functional overlap, but rather an idiosyncratic contribution to ecosystem functioning. However, we hypothesize that functional redundancy is more likely to observe when taking into account the entire diversity of natural assemblages. We conducted a microcosm experiment with two natural communities to assess their stress response to elevated temperature. The two communities differed in diversity (high [HD] vs. low [LD]) and environmental origin (harsh vs. moderate conditions). We assessed their stress resistance to the experimental treatment in terms of species and diversity changes, and their function in terms of abundance, biomass, and trophic diversity. According to the Insurance Hypothesis, we hypothesized that the HD community would cope better with the stressful treatment due to species functional overlap, whereas the LD community functioning would benefit from species better adapted to harsh conditions. Our results indicate no evidence of functional redundancy in the studied nematofaunal communities. The species loss was more prominent and size specific in the HD; large predators and omnivores were lost, which may have important consequences for the benthic food web. Yet, we found evidence for alternative diversity–ecosystem functioning relationships, such as the Rivets and the Idiosyncrasy Model.

## Introduction

Biodiversity has diminished dramatically over the past decades with climate change being among the main responsible factors [1], [2]. Increasing sea surface temperature is one of the consequences of climate change, and possibly the most pervasive of present-day impacts on marine systems [3]. In intertidal areas, the combination of high water-temperature and long exposure to high air-temperature during spring tides may exceed the tolerance of some intertidal organisms causing local extinctions [1]. Ecological services provided by intertidal organisms such as water filtration, nutrient recycling and sediment stabilization [4], [5], [6] may be at risk due to changes in community patterns and the loss of species.

Free-living marine nematodes are usually the most abundant and diverse benthic meiofaunal taxon in marine sediments. They comprise a variety of feeding guilds including bacteria- and diatom-feeding species, scavengers and predators, which play a fundamental role in the benthic food web as recyclers and as a trophic link between microorganisms and macrofauna [7], [8], [9], [10], [11]. Additionally, recent studies have revealed their importance for sediment stability, as their presence enhances the production of sediment-binding extracellular polymeric substances (EPS) [6]. Their communities are characterized by an exceptionally high taxonomic and functional diversity. According to the Insurance Hypothesis (IH) [12], diverse communities benefit from the greater potential redundancy of similar species, as it may assure ecosystem functioning following considerable species loss caused by environmental hazards. Although such functional redundancy has been observed in microbial communities [13], benthic macro-invertebrate species seem to contribute idiosyncratically to ecosystem functioning with their impact strongly depending on species identity and functional role [14], [15], [16], [17], [18]. Whether this also applies to benthic meiofauna remains to be investigated. Although meiobenthic assemblages typically comprise many seemingly functionally similar species [4], [19], experimental results on selected bacterivore species suggest that functional overlap within established guilds – with functional diversity most often approximated by the diversity in feeding groups – may be smaller than expected, resulting in an idiosyncratic relationship between diversity and ecosystem functioning [4].

To date, the subject of functional redundancy in nematodes has been investigated on experimental systems that included very few functionally similar bacterivore species [4], [20]. In these low-diversity assemblages, each species contributed idiosyncratically. However, studies addressing species redundancy in natural nematode assemblages are lacking but necessary to assess the complexity of community responses to environmental forcing. Nematode assemblages are characterized by high local as well as within-guild diversity; in line with the insurance effect, we hypothesize that functional redundancy is likely more prominent in assemblages of higher diversity, where it may buffer nematode functional roles against perturbations. Therefore, we conducted a microcosm experiment with natural communities drawn directly from their environment to assess their stress response to elevated temperature. We assessed their stress resistance to the experimental treatment in terms of species and diversity changes, and their function in terms of abundance, individual and community biomass, and trophic diversity. The communities originate from a macrotidal ridge-and-runnel beach of the northern Gulf of California, Mexico, one of the world’s marine endemism and biodiversity hotspots [21]. This semi-enclosed sea is exceptionally prone to sea temperature increase (8°C over the past century [22]), putting at stake a high number of marine species [23]. Based on a previous study on the same beach conducted in 2007/2008 [24], we chose two different assemblages stemming from different environments: a low-diversity community from the upper beach (LD henceforth), where environmental conditions are rather harsh (long tidal exposure and high temperature), and a high-diversity (HD henceforth) community from a lower part of the beach, where environmental conditions are comparatively moderate (relatively shorter tidal exposure and on average lower temperature). We hypothesized that a) the less diverse community would largely consist of species, which cope better with stress and hence would lose relatively fewer species because of its environmental history of high temperature and long exposure during low tide. However, according to the IH, we also hypothesized that b) the HD community would better maintain its functionality in the face of environmental stress due to the compensatory potential of functionally redundant species, and/or due to the higher probability that high-diversity assemblages contain high-performance or resistant species (i.e. sampling effect) [4].

We therefore expected that the imposed temperature stress would, on the one hand, drive the HD and LD communities to structural convergence based on a set of temperature-resistant species; and, on the other, that the HD assemblage would exhibit a higher degree of functionality than the LD after an environmentally stressful phase, as predicted by the IH.

## Materials and Methods

### 1. Rationale behind the choice of sampling sites and the proxies to assess ecosystem functioning

The rationale behind the choice of the two sampling sites was to select two assemblages exhibiting different diversity levels (*i.e.*, species richness and Shannon’s diversity index), yet no radical taxonomical differences such that diversity *per se* would be confounded with the taxonomic composition of the community. Moreover, the ecological background should not be radically different so that the ecological difference would not be the main driver of the response to increased temperature. For this reason we chose two stations located on different sandbars in the upper half of the middle beach.

The functioning of the system was assessed with several proxies that integrate different aspects of nematode functionality: abundance, individual and community biomass, and trophic diversity. Abundance together with individual and community biomass are indicators of survival, population turnover rate, and secondary production, which in turn are linked to, e.g., community respiration (higher abundance/biomass  =  higher respiration rate), nutrient uptake rate (higher abundance/biomass  =  higher nutrient uptake rate) and ultimately also to nutrient regeneration. Given that larger nematodes move more easily in the sediments [25] individual biomass may also be an indicator of micro-bioturbation with implications for the porosity of the sediment and therefore chemical processes and ultimately nutrient regeneration. The trophic identity and community trophic diversity are directly linked to the nematodes feeding habits and therefore available food sources in the system, which, in turn, reflect, e.g., the nutrient regeneration and transfer to higher trophic levels. Our functional proxies are thus relevant for a broad range of nematode functions, although functional overlap may vary among the different functions.

### 2. Sampling site and strategy

**Figure 1:**
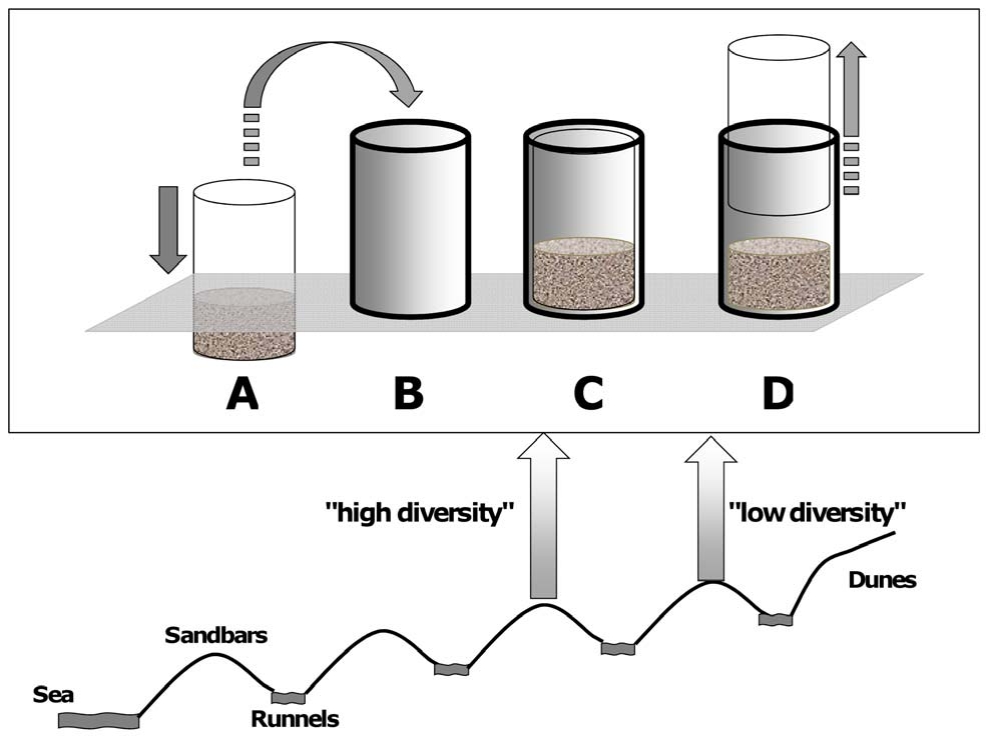
Sampling strategy in the field. 40 sediment cores were sampled at two stations (20 cores each) located on different sandbars of the intertidal, and hosting communities of high or low diversities respectively. A: Samples were taken with a metal cylinder to 10 cm depth. B: Entire sediment cores were placed into containers. C: The sampling corer fitted exactly in the container so that the internal structure of the sediment core remained as intact as possible. D: The metal corer was carefully removed.

Samples were taken on September 1^st^ 2008 during low spring tide at *El Tornillal* (N: 31° 33.527; W: 114° 17.866) in the Upper Gulf of California, Mexico (UGC). *El Tornillal* is a 600 m wide dissipative ridge-and-runnel macrotidal beach [26]. For a more detailed description of the study site we refer to Gingold et al. (2010) [24]. We chose two stations based on previous knowledge of the beach [24], located approximately 150 m away from each other. The station closer to the waterline (ca. 350 m from the waterline, ca. 2.2 m above mean lower low water level [MLLW]) was the "high diversity" (HD) site, and the one closer to the dunes (ca. 500 m from the waterline, ca. 2.9 m above MLLW) the "low diversity" (LD) site (Figs. 1 and S1). Daily mean tidal exposure to air for the month of sampling were 15.6 h for the LD site, and 11.7 h for the HD site (Fig. S2). Differences in taxonomic diversity between sites were verified at the time of sampling under a Leica Zoom 2000 stereoscope. Twenty sediment cores were taken at equal distances within 20 m^2^ to a depth of 10 cm at each of the two sites with a metal corer (10.8 cm in diameter) and carefully transferred to microcosm containers (Fig. 1A–D). All microcosms were immediately placed in cooler-boxes filled with tempered seawater and brought as fast as possible to the aquaculture facilities at the Centro de Investigación Científica y Educación Superior de Ensenada (CICESE).

In addition to the microcosm cores, four sediment samples were taken at each site (LD and HD) with a small corer (9.8 cm long by 2.9 cm in diameter) to evaluate the nematode community composition and diversity in the field at the time of sampling. These samples were fixed immediately in 5% formalin. In order to characterize the habitat of both locations, four replicate samples were taken for each of the following analyses: 1) granulometry, 2) organic matter and 3) chlorophyll-*a* as a proxy for microphytobenthos biomass. Core size for granulometry and organic matter was 9.8 cm long by 2.9 cm in diameter, and for microphytobenthos 1 cm long by 1 cm in diameter. Granulometry and organic matter samples were kept under ice in the field, and then frozen at –20°C until processed. Chlorophyll samples were kept in dark tubes under ice in the field, and then stored at –40°C until processed. Water temperature at the time of sampling was 31°C.

### 3. Experimental setup

In the laboratory, four microcosms (two HD and two LD respectively) were randomly assigned to each of ten 100 l tubs. Previously, the tubs had been filled with filtered and sterile seawater (passed through a 1 µm filter and UV treated) to minimize biofouling and maintained at 31°C. All microcosms were acclimated to experimental conditions at 31°C for 5 days. Temperature was maintained constant with thermostats (1000 W titanium heater for high, and regular 250 W heaters for the normal temperature), and the water was homogenized with bubbling air stones placed next to the heaters. All microcosm containers were closed with a transparent plastic lid. Water oxygenation and circulation inside the microcosm containers was achieved by bubbling air with an air stone introduced through a hole in the lid.

**Figure 2:**
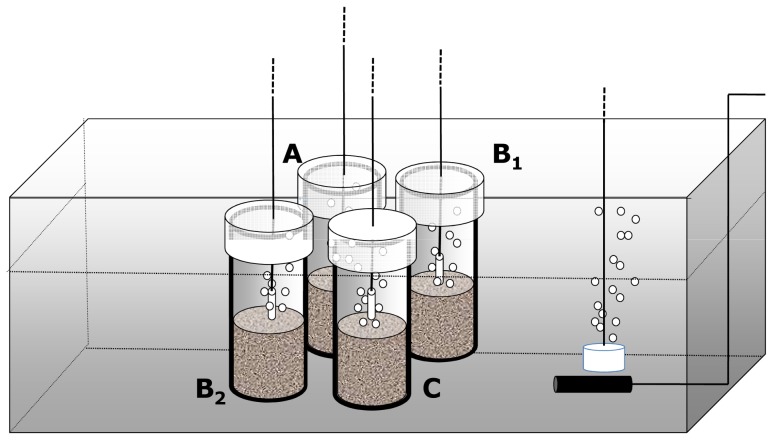
Experimental design. 4 microcosm containers were placed in each of 10 tubs. The time_0_ control (A) was analyzed at the start of the experimental treatment when temperature was changed in the high temperature treatments. The experimental groups of high (B_1_) and low (B_2_) diversity were kept at constant temperature throughout the experiment: elevated (36°C) for the test group and normal (31°C) for the "temperature control" group. Each experimental group was replicated 5 times. The live control (C) was used to monitor nematodes throughout the experiment.

After the acclimation period, the temperature was gradually (during 24 h) increased to 36°C in five randomly assigned high temperature tubs. We chose a relatively short acclimation period in order to start the experiment under conditions as close as possible to the original state, based on the assumption that temperature acclimation of nematodes is expected to be relatively fast (order of hours [27]). The experimental temperature (36°C) is above the highest recorded mean summer temperature (31.1°C±1°C) but within the range of future (50–100 years) temperatures that could be reached following linear extrapolations of *in situ* temperatures over the past 40 years (M. Lavín, pers. comm.). As soon as the temperature reached 36°C, one of the four containers was removed from each tub (five replicates from each HD and LD group respectively). These microcosms represented the "time zero control" (t_0_) at the start of the experimental treatment and served as a control for the enclosure effect by the microcosm device (Fig. 2A). At the end of the experiment, two microcosms were sampled representing HD and LD respectively (Fig. 2B_1_ and B_2_). The remaining microcosm (one in each tub) served as a "live control" to continuously monitor and record activity of nematodes during the course of the incubation (Fig. 2C). Each of these ten live controls was sub-sampled once at regular intervals (4 days) from the acclimation period to the end of the experiment. The microcosms themselves were left in the tubs after sub-sampling. Sampled organisms were immediately checked under a light stereoscope.

The experimental treatment consisted of high temperature incubation (maintained at 36°C), whereas the experimental temperature control was incubated at field temperature (maintained at 31°C). Henceforth, abbreviations of the different groups (*i.e.*, diversity levels and treatments) will be used according to Table 1. Microcosms were checked daily and constant salinity was maintained (ca. 35‰), compensating evaporation by adding reverse osmosis purified water. Tubs were refilled daily to maintain a constant water level. The experiment was run for 25 days, after 5 days of acclimation and 1 of gradual temperature change. Through the regular checks of the "live controls" a continuous mortality in both treatments could be observed. The experiment was stopped at the time when anoxic conditions were first detected in the sediments of the high temperature treatment, together with decreased activity of the nematodes in both treatments. Despite the anoxic conditions at the end of the experiment, we are confident that any effect of anoxia/hypoxia is likely to be subordinate to the temperature-induced effects, as no sudden collapse of microcosms in either treatment could be observed. At the end of the experiment, whole microcosms were fixed in 5% formalin.

**Table 1:** Abbreviations for experimental groups.

	Low diversity	High diversity
**Time zero control** 1	LDt_0_	HDt_0_
**Normal temperature**	LD_31_	HD_31_
**High temperature**	LD_36_	HD_36_

1 Samples taken at the time when high temperature treatments reached their target temperature (36°C).

### 4. Faunal analyses

Fixed samples were rinsed with freshwater over a 45 mm sieve. Meiofauna was extracted by suspension in colloidal silica (LUDOX™, specific density 1.15) following De Jonge & Bouwman [28] and stored in 80 ml 5% formalin. Nematodes were counted in five aliquots of 5 ml (25 ml in total per sample) using a counting dish under a Leica Zoom 2000 stereoscope. Nematode density (ind. 10 cm^−2^) was calculated by the mean abundance of the five aliquots and extrapolated to total abundance based on the fraction (31.25%) of the volume of each aliquot relative to the total sample. All aliquots were transferred to a 5% glycerol solution and slowly evaporated on a heating plate. The first 100 randomly picked nematodes were mounted on permanent slides for identification using an OLYMPUS BX51 compound microscope with differential interference contrast (DIC) optics. Nematodes were identified to the species or genus level where possible, using pictorial keys [29], [30], [31]. In the case where male reproductive organs were essential to determine the genus, juveniles and females were determined in the most conservative way: if possible, they were identified to the generic level; alternatively they were identified to family level. If species could not be determined, they were labeled sp. 1, sp. 2.

The community attributes (response variables) tested were the number of species (species richness, *S*), the taxonomic diversity (Diversity index of Shannon Wiener, *H’*) and the number of individuals per 10 cm^2^ (abundance) to assess the structural changes. The index of Shannon Wiener was calculated as *H'* 
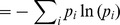
, where *p_i_* is the proportion of the total count arising from the *i*th species. In order to evaluate functional changes in the system, we estimated trophic diversity (Index of trophic diversity, *ITD^−1^*) and biomass (individual and community).

Trophic diversity was assessed based on the four trophic groups according to Wieser [32]: 1A (selective deposit and bacteria feeders with small, unarmed buccal cavities), 1B (non-selective deposit feeders with unarmed wide buccal cavities), 2A (epistrate feeders with lightly armed small buccal cavities) and 2B (carnivores and omnivores with wide armed buccal cavities). Although the trophic groups are based on morphological features rather than direct foraging observations, they are assumed to be directly linked to the potential feeding behavior. The trophic diversity is calculated by an index (Index of trophic diversity, ITD) modified from Heip et al. [33] applying the formula 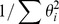, where θ*_i_* is the fraction of the *i*th functional group. It is therefore presented as *ITD^−1^*. *ITD^−1^* ranges from 1 (when one functional group contributes 100% and functional diversity is lowest) to 4 (when each functional group contributes 25% and functional diversity is highest). A functionally diverse assemblage is thus characterized by the even representation of the four trophic groups. The method allows for an approximate assessment of functional overlap when two species belong to the same trophic guild.

Pictures were taken of all individuals for morphometric and biomass analyses. Biomass serves as an indirect proxy for secondary production. Direct measurement of secondary production of nematodes in natural assemblages is hampered by the fact that many species have continuous reproduction and overlapping generations. Hence, biomass is often used as a surrogate for secondary production, as it is directly proportional to community respiration [33], [34], [35]. Total body length and body width of each of the 100 individual nematodes per sample were measured using the software ImageJ. Individual biomass for each nematode was then calculated following a slightly adapted version of Andrassy’s formula [36]: Biomass (in µg wet weight)  =  (*LW* ^2^/1.7) *NRd* * 10^3^, where *L*  =  total body length (mm), *W*  =  body width (body diameter in mm), *NRd* =  relative density, estimated at 1.13 for marine nematodes [37]. Community biomass (in µg wet weight) was calculated as the average individual biomass*number of individuals (abundance).

### 5. Habitat characterization of the sampling stations

Sediment granulometry was assessed by the dry sieving method. Samples were first treated with 30% peroxide (H_2_O_2_) to oxidize organic matter. After rinsing gently with distilled water and drying at 60° C they were sieved through a stack of Wentworth grade sieves (0, 0.5, 1, 1.5, 2, 2.5, 3, 3.5 and 4 Φ, where Φ  =  –log_2_ [grain diameter]) and the dry weight of each fraction was obtained [38].

Organic matter content was assessed by combustion at 550° C for 24 hours, after treating samples with 10% HCl to dissolve inorganic carbonates (mainly CaCO_3_ [39]), rinsing them thoroughly with fresh water, and lyophilization [40]. Organic matter was computed as the difference in dry weight before and after combustion and standardized to percentage of total dry weight before combustion.

Phytobenthic chlorophyll was extracted by grinding sediment samples in 90% acetone, extracting for 24 hrs in the dark and then centrifuging at 3,000 rpm for 10 minutes. Absorbance of the supernatant was measured at 665 and 750 nm before and after acidification with a few drops of 10% HCl (Spectrophotometer Ely-2000, Elyptica, Ensenada B.C.). Chlorophyll density was calculated following Lorenzen [41] and Colijn & Dijkema [42] and is expressed in mg m^−2^.

### 6. Statistical analyses

To analyze differences in nematode assemblages among experimental groups we applied Analyses of Similarity (ANOSIM) on Bray-Curtis similarities. ANOSIM is conceptually similar to ANOVA but makes no assumptions about the distribution of the data. The test statistic R  =  1 if all replicates within groups are more similar to each other than to any replicate from different groups, whereas R  =  0 if similarities within and among groups are the same on average. Analysis of Similarity Percentage (SIMPER) was applied to assess the species that contributed the most (*i.e.*, were the most "typical") to each of the assemblages. To visualize the relative contribution of the characteristic species of each experimental group, we plotted results from SIMPER (90% cumulative percentage) in a doughnut chart.

We used Student’s t-tests to assess differences in community and environmental attributes between the two field stations. Assumptions of normal distribution and homoscedasticity were met as verified by a Kolmogorov-Smirnov and Bartlett’s test respectively.

Differences in community attributes of experimental groups were tested with ANOVAs. The main predictions of this study relate to the independent effect of temperature in each diversity group, therefore we made an *a priori* test of independence of temperature (time_0_, 31°C and 36°C) and diversity (high and low) on response variables with a 3×2 factorial Type II ANOVA. Then, we applied 1-way ANOVA separately to test the null hypotheses of no differences between the mean response variables of the time_0_ control and the different treatments (*H_0_*: m-*x*Dt_0_  =  m-*x*Dt_31_  =  m-*x*Dt_36_), where m is the mean response variable of each diversity treatment, and *x* refers to L (low diversity) or H (high diversity). Rejection of *H_0_* was further investigated using Dunnett test for multiple comparisons [43], taking *x*Dt_0_ as the control group. Rejection of *H_0_*: m-*x*Dt_0_  =  m-*x*Dt_36_ was interpreted as evidence of a significant high temperature effect with or without significant enclosure effect, the latter being assessed by the rejection of H_0_: m-*x*Dt_0_  =  m-*x*Dt_31_. If both effects were significant, t-tests between xD_31_ and xD_36_ were performed in order to assess whether both effects were additive (in case of significance) or whether the enclosure effect was dominant (in case of non-significance). Homoscedasticity was verified with Bartlett’s test and normality with the Kolmogorov-Smirnov test [44]. In the case of heteroscedasticity data were log-transformed.

PRIMER version 6 [45], [46] was used for multivariate analyses. STATISTICA [47] was used for univariate analyses.

### 7. Ethics statement

Samples were collected under permit No. DGOPA. 05335.100707.2458 (SAGARPA).

## Results

### 1. Biological and environmental differences between source communities in the field

**Figure 3:**
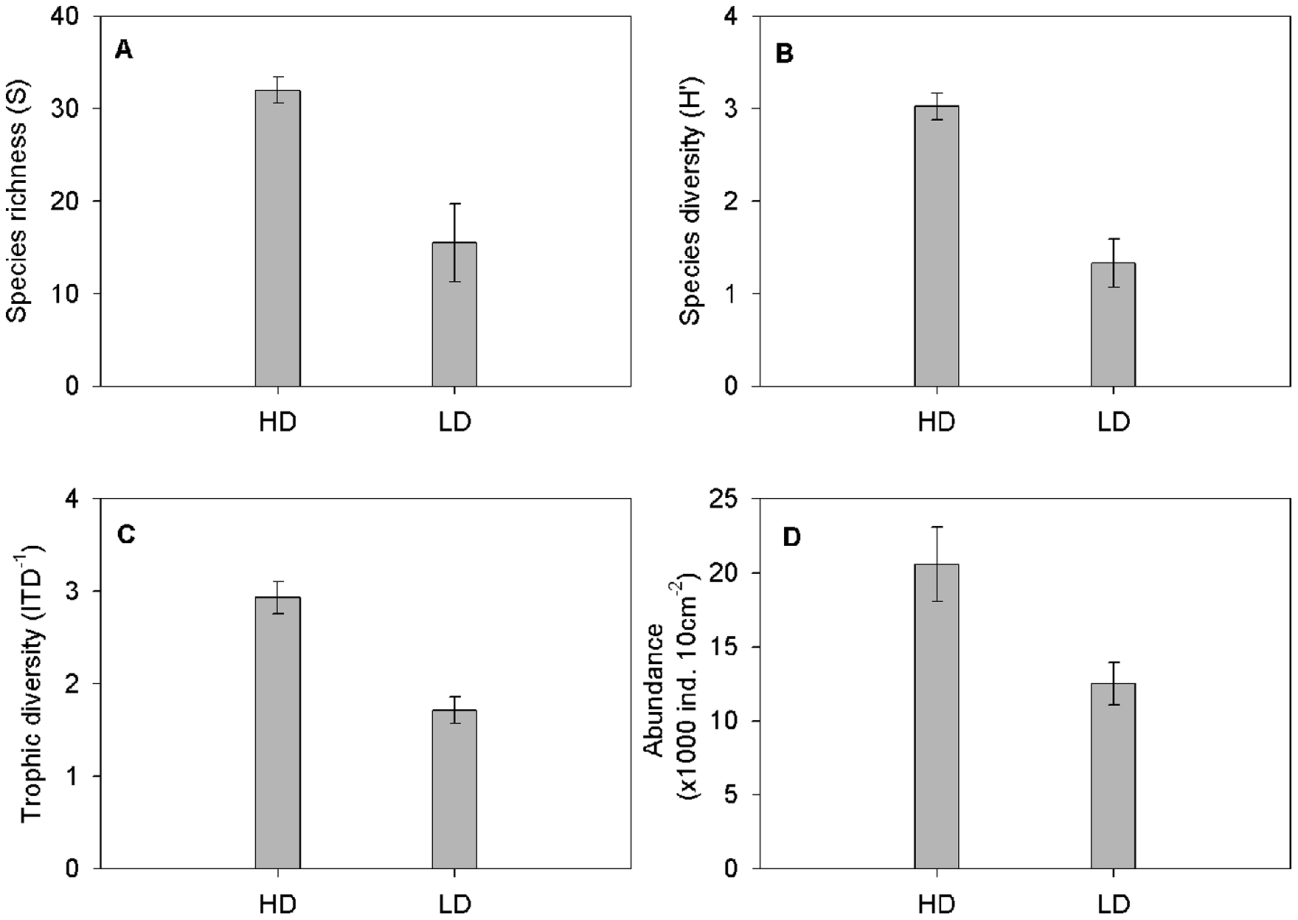
Comparison of the two field assemblages. Mean (± Standard deviation) of A: Species richness (*S*), B: Diversity (*H’*; Index of Shannon Wiener), C: Trophic diversity (*IDT^−1^*), and D: Abundance (individuals per 10 cm^2^).

The two sampled stations hosted different nematode assemblages (ANOSIM, R = 1, p = 0.029); the HD assemblage had significantly higher species richness, diversity (*H’*), trophic diversity (*ITD^−1^*), and abundance (individuals per 10 cm^2^) (Table 2, Fig. 3A-D).

**Table 2:** Student’s t-test of community attributes between the two field sampling stations (n = 4).

	t	p
**Species richness**	7.44	<0.001
**Diversity** 1	11.28	<0.0001
**Abundance**	5.55	0.002
**Trophic diversity** 2	11.04	<0.0001

1 Shannon Wiener diversity index.

2 Index of trophic diversity ITD^−1^.

**Figure 4:**
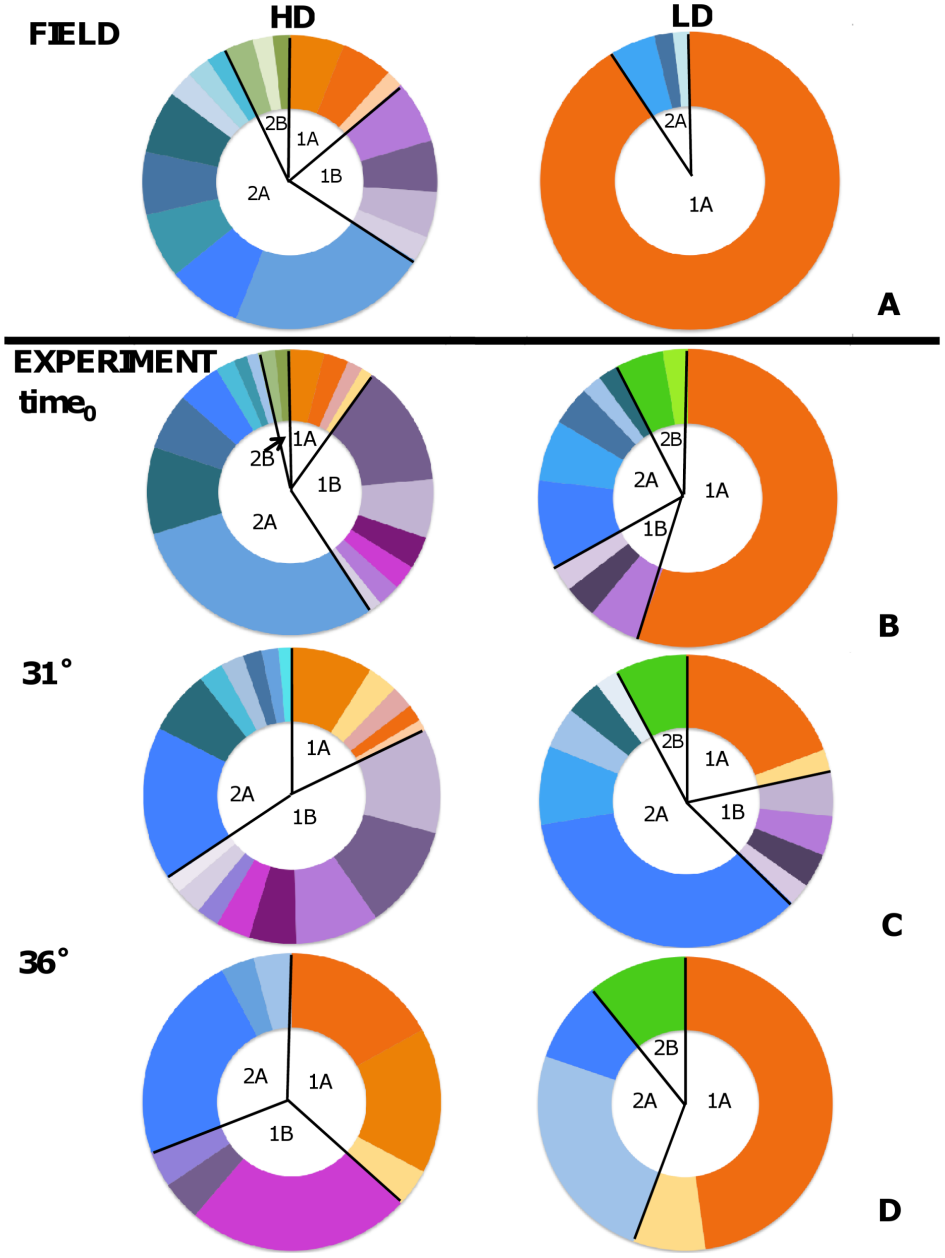
Graphical representation of typical species of each diversity group. Species are represented by different colors. The corresponding percentage (up to 90%) of contribution was calculated by SIMPER and is listed in Table 3. HD = High diversity, LD = low diversity. A: The two communities sampled in the field, B: Experimental control groups (before the start of the experiment), C: Assemblages exposed to normal temperature and D: Assemblages exposed to high temperatures.

SIMPER analysis revealed that the LD assemblage was largely dominated by *Perepsilonema* sp. (83.61%, Fig. 4A, Table 3). Considering all species (and not only the cumulative 90% represented in the SIMPER analysis), the two assemblages shared 20 species. In addition, 12% of all species were unique to the LD whereas 31% were unique to the HD. *Perepsilonema* sp., *Microlaimus* sp.2, *Metachromadora* sp.2 and *Chromadorina* sp., making up 90% of the discriminating species of the LD (*i.e.*, being the most typical species), were also present at the HD with the exception of *Chromadorina* sp. (*Microlaimus* sp.2 was not part of the discriminating species shown in Table 3). Thus, the large difference between the two assemblages revealed by ANOSIM is mainly due to a) the dominance of a single species (*Perepsilonema sp.* contributed almost 40% to the dissimilarity between the two assemblages) and b) the large number of unique species in the HD.

**Table 3:** Percentages contribution (%) of the top 90% discriminating genera of the two field stations and the six experimental groups.

FIELD					
	HD^1^	%		LD^2^	%
**1A** 3	*Tricompa* sp.1	5.38	**1A**	*Perepsilonema* sp.	83.61
	*Perepsilonema* sp.	5.1	**2A**	*Microlaimus* sp.2	4.36
	*Ceramonema* sp.2	1.7		*Metachromadora* sp.2	1.86
**1B**	*Rhynchonema* sp.	6.23		*Chromadorina* sp.	1.66
	*Xyala* sp.2	5.1			
	Xyala sp.1	4.53			
	*Omicronema* sp.	2.55			
**2A**	*Metachromadora* sp.1	19.83			
	*Desmodora* sp.1	7.37			
	*Chromadorita* sp.1	6.51			
	*Metachromadora* sp.2	6.23			
	*Pomponema* sp.	6.24			
	*Dichromadora* sp.	2.55			
	*Hypodontolaimus* sp.	2.27			
	*Chromaspirinia* sp.	1.98			
**2B**	*Epacanthion* sp.	2.83			
	*Odontophora* sp.	1.99			
	*Enoploides* sp.	1.69			

EXPERIMENT					
Time_0_	HD	%		LD	%
**1A**	*Tricoma* sp. 1	3.5	**1A**	*Perepsilonema* sp.	50
	*Perepsilonema* sp.	2.53	**1B**	*Rhynchonema sp.*	5
	*Calomicrolaimus*	1.56		*Theristus* sp.2	3.23
	*Ceramonema* sp.3	1.17		*Theristus* sp.1	2.58
**1B**	*Xyala* sp.2	12.65	**2A**	*Desmodora* sp.1	8.55
	*Xyala* sp.1	6.03		*Microlaimus* sp.2	5.97
	*Xyalidae* gen.1	3.31		*Metachromadora* sp.2	3.87
	*Richtersia* sp.	2.53		*Microlaimus* sp.1	1.94
	*Rhynchonema* sp.	2.33		*Pomponema* sp.	1.94
	*Omicronema* sp.	1.36	**2B**	*Gammanema* sp.	4.68
**2A**	*Metachromadora* sp.1	26.85		*Adoncholaimus* sp.	2.42
	*Pomponema* sp.	8.95			
	*Metachromadora* sp.2	5.84			
	*Desmodora* sp.1	4.47			
	*Chromaspirinia* sp.	1.95			
	*Chromadorita* sp.1	1.36			
	*Microlaimus* sp.1	1.17			
**2B**	*Epacanthion* sp.	1.75			
	*Enoploides* sp.	1.56			

Normal T (31°)	HD^1^	%		LD^2^	%
**1A**	*Tricoma* sp.1	8.01	**1A**	*Perepsilonema* sp.	17.77
	*Ceramonema* sp.3	2.98		*Ceramonema* sp.3	2.26
	*Calomicrolaimus* sp.	2.23	**1B**	*Xyala* sp.1	4.53
	*Perepsilonema* sp.	1.68		*Rhynchonema* sp.	4.01
	*Ceramonema* sp.2	1.12		*Theristus* sp.2	3.48
**1B**	*Xyala* sp.1	10.24		*Theristus* sp.1	2.26
	*Xyala* sp.2	10.24	**2A**	*Desmodora* sp.1	32.4
	*Rhynchonema*	8.19		*Microlaimus* sp.2	8.01
	*Xyalidae* gen.1	4.66		*Microlaimus* sp.1	4.18
	*Richtersia* sp.	3.35		*Pomponema* sp.	3.66
	*Sabatieria* sp.	2.23		*Paracyatholaimus* sp.	2.26
	*Omicronema* sp.	2.61	**2B**	*Gammanema* sp.	7.32
	*Stylotheristus* sp.	1.68			
**2A**	*Desmodora* sp.1	15.27			
	*Pomponema* sp.	6.33			
	*Chromaspirinia* sp.	2.42			
	*Kraspedonema* sp.	2.23			
	*Metachromadora* sp.2	1.86			
	*Metachromadora* sp.1	1.68			
	*Spirinia* sp.1	1.3			

High T (36°)	HD^1^	%		LD^2^	%
**1A**	*Perepsilonema* sp.	15.52	**1A**	*Perepsilonema* sp.	43.96
	*Tricoma* sp.1	14.48		*Ceramonema* sp.3	7.25
	*Ceramonema* sp.3	3.79	**2A**	*Microlaimus* sp.1	22.54
**1B**	*Richtersia* sp.	22.24		*Desmodora* sp.1	8.21
	*Xyala* sp.2	3.97	**2B**	*Gammanema* sp.	9.98
	*Sabatieria* sp.	3.62			
**2A**	*Desmodora* sp.1	20.86			
	*Metachromadora* sp.1	3.28			
	*Microlaimus* sp.1	3.79			

1 HD = High diversity.

2 LD = Low diversity.

3 1A, 1B, 2A, 2B  =  Feeding groups according to the classification of Wieser (1953).

Trophic diversity was lower in the LD: nematodes accounting for 90% of the cumulative similarity only comprised two trophic groups, namely selective deposit feeders (1A) and epistrate feeders (2A). The predominance of selective deposit feeders was again due to the predominance of *Perepsilonema sp*. (Table 3).

Environmentally, the two stations differed slightly but significantly only in sediment mean grain size, but not in sediment organic matter or chlorophyll*-a* content (Table 4).

**Table 4:** Student’s t-test of environmental variables between the two field sampling stations (n = 4).

	t	p
**Mean grain size**	10.64	<0.0001
**Organic matter content**	1.95	0.1
**Chlorophyll** ***-a***	–0.0006	0.99

### 2. Initial conditions of experimental units (*x*Dt_0_)

**Figure 5:**
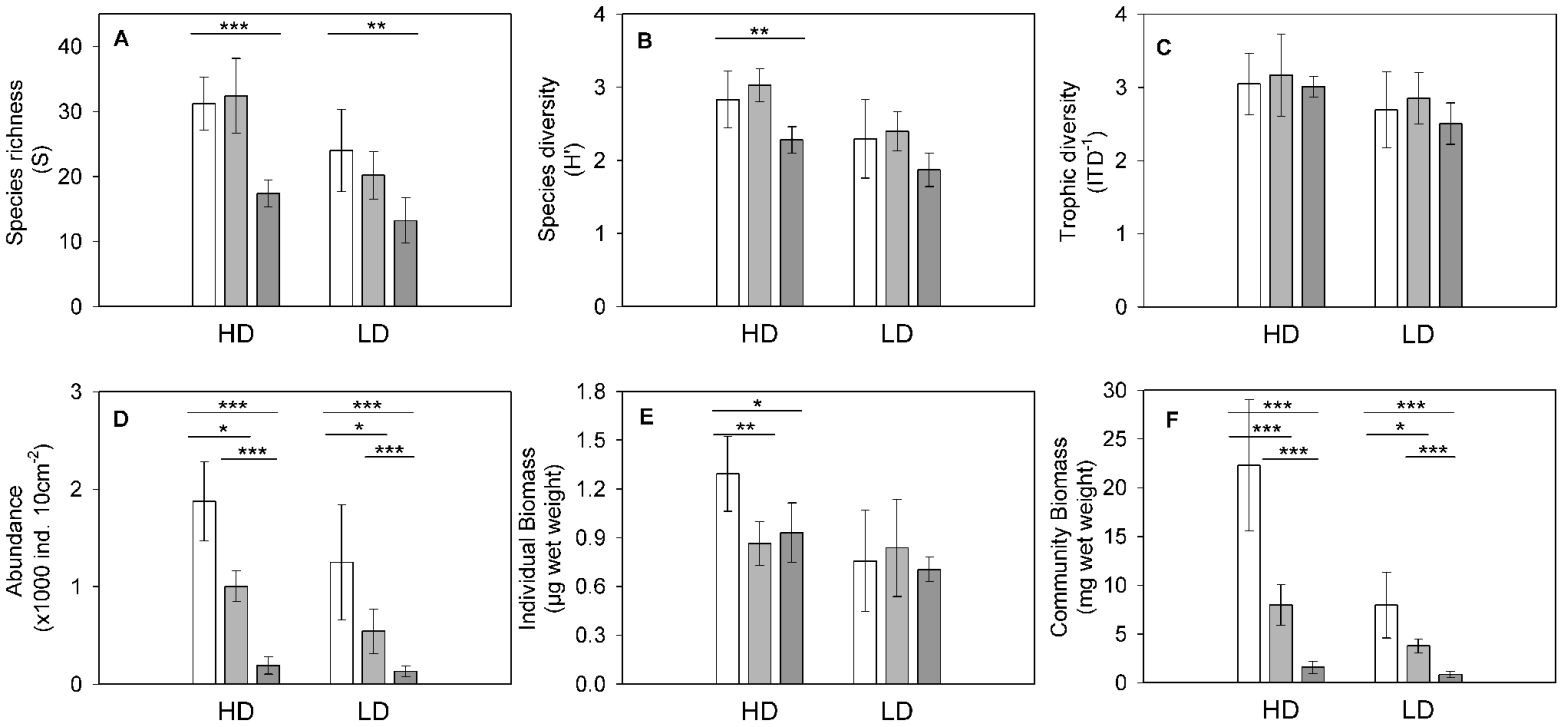
Comparison of the experimental assemblages. Mean (± standard deviation) of A: Species richness (*S*), B: Diversity (*H’*; Index of Shannon Wiener), C: Abundance (individuals per 10 cm^−2^), D: Trophic diversity (*IDT^−1^*), E: Individual biomass (in µg wet weight), and F: Community biomass (in mg wet weight). HD  =  High diversity, LD  =  Low diversity. White bars  =  time_0_ controls, light gray bars  =  control temperature (31°C), dark gray bars  =  high temperature (36°C). Asterisks indicate significance levels after multiple comparisons with Dunnett test between xDt_0_ and xD_31_ and xD_36_ respectively. Significance between xD_31_ and xD_36_ were assessed with Student’s t-tests. *<0.05, **<0.01, ***<0.001.

At the start of the experimental treatment, the two assemblages (HDt_0_ and LDt_0_) were significantly different (ANOSIM R =  0.972, p = 0.008). More species (19) contributed to the 90% cumulative similarity of the HDt_0_ compared to the LDt_0_ (11; Fig. 4B, Table 3). The HDt_0_ hosted a total of 57 species and more unique species (24) than the LDt_0_ (total: 46, unique: 13). The differences for species richness, diversity (*H’*), abundance and trophic diversity (*ITD^−1^*) (Fig. 5A–D), were less pronounced than those observed in the field. A larger fraction of large nematodes in the HD assemblage accounted for the higher individual and community biomass (Fig. 5E and F).

### 3. Structural changes in community resulting from high temperature effects: species richness and diversity

Temperature and diversity acted independently on structural attributes of the community as indicated by the non-significant interaction effect (3×2 factorial ANOVA, Table 5). High temperature had a clearly negative effect on species richness in both HD and LD assemblages (Fig. 5A). Non-significant differences in *S* and *H’* between t_0_ and t_31_ microcosms both in HD and LD revealed that significant differences between t_0_ and t_36_ were attributable to high temperature exposure. Significantly less species survived the high temperature exposure in both LD_36_ and HD_36_ relative to their t_0_ controls (Table 6, Fig. 5A). Consequently, these microcosms exhibited decreased levels of diversity (*H’*) at the end of the 25 days of incubation, although the change was significant only in HD (Table 6, Fig. 5B). The enclosure effect on species richness or H’ was not significant in the HD or LD group (Fig. 5B). The dissimilarity of the assemblages caused by the high temperature was much higher in the HD group (HDt_0_ - HD_36_: 70.08) than in the LD group (LDt_0_ - LD_36_: 52.04) as revealed by SIMPER. In fact, in both LD and HD, the dissimilarity caused by the enclosure effect was in a similar range as the temperature effect in LD (HDt_0_ – HD_31_: 53.44; LDt_0_ – LD_31_: 51.48).

**Table 5:** F- and p-values of 2×3 factorial ANOVA (n = 5).

	Species richness		Shannon diversity		ITD		Ind. biomass		Abundance		Community biomass	
	**F**	**p**	**F**	**p**	**F**	**p**	**F**	**p**	**F**	**p**	**F**	**p**
**T**	22.9	0.00	10.4	0.00	1.0	0.40	2.5	0.10	91.3	0.00	127.9	0.00
**D**	23.3	0.00	19.4	0.00	7.1	0.01	10.5	0.00	12.3	0.00	40.1	0.00
T*D	2.1	0.15	0.3	0.76	0.2	0.86	3.4	0.05	0.4	0.67	1.1	0.36

Factors consist of temperature (T, three levels: time_0_, 31° and 36°) and diversity (D, two levels: high and low).

**Table 6:** F- and p-values of 1-way ANOVAs for each diversity group (n = 5).

	High diversity		Low diversity	
	F	p	F	p
**Species richness**	19.2	0.0002	6.9	0.01
**H' diversity**	9.7	0.003	2.9	0.096
**ITD^−1^**	0.2	0.82	0.9	0.41
**Abundance**	65.4	<0.0001	34.0	<0.0001
**Individual Biomass**	7.6	0.007	0.4	0.71
**Community biomass**	85.0	<0.0001	48.3	<0.0001

The factor ( = treatment) consists of three levels: time_0_, 31° and 36°.

A few taxa became more dominant under the temperature stress treatment (Fig. 4D, Table 3). *Perepsilonema* sp., *Tricoma* sp., *Richtersia* sp., *Desmodora* sp.1 and *Metachromadora* sp.1 increased in abundance in HD_36_. *Sabatieria* sp. also increased in HD_31_ and HD_36_ appearing for the first time among the top 90% discriminating species (Fig. 4C and D, Table 3). *Microlaimus* sp.1 and *Gammanema* sp. increased in the LD_36_, and *Ceramonema* sp.3 appeared in LD_31_ and LD_36_ among the 90% discriminating species (Fig. 4C and D, Table 3).

### 4. Functional changes in community resulting from high temperature and enclosure effects: abundance, individual and community biomass and trophic diversity

The temperature treatment acted independently from the diversity level (HD or LD) on the functional attributes of the community (3×2 factorial ANOVA, Table 5). Abundance and community biomass decreased significantly in both, HD_36_ and LD_36_, due to the combined effects of temperature and experimental enclosure (Fig. 5D and F, Table 6). The loss of individuals was species specific, since the high temperature also caused a significant loss of species richness (in both, HD and LD) and diversity (in HD). Decreased abundance in the HD due to the enclosure effect was also size-specific, and as a consequence, mean individual biomass decreased in response to the experimental enclosure effect as well. For instance, two of the four species lost exclusively due to the enclosure effect in HD, namely *Epacanthion* sp. and *Enoploides* sp., were among the largest in this study and contributed to, but were not exclusively responsible for, the significant loss of individual biomass. By contrast, the loss of 6 species in the LD was not size specific, since mean individual biomass remained unaffected.

The functional diversity of both the HD_36_ and LD_36_ communities was affected to a large extent as they suffered the loss of an entire, yet different trophic guild. The HD community lost trophic group 2B consisting of large-sized predators and omnivores due to enclosure and temperature effects (Fig. 4C and D, Table 3). By contrast, the decrease in abundance of the LD_36_ microcosms was related to the loss of trophic group 1B consisting of size-unspecific, unselective deposit feeders due to the temperature effect only (Fig. 4D, Table 3). Even though both assemblages lost an entire, yet different trophic group, these changes were not reflected in significant *ITD^−1^* differences (Fig. 5C, Table 6). The two species representing 2B in HDt_0_ (*Epacanthion* sp. and *Enoploides* sp.) disappeared due to the enclosure effect. This is consistent with the loss of individuals (*i.e.*, decreased abundance) due to the combined enclosure and temperature effect. In the low diversity microcosms, of the two species representing 2B in LDt_0_ (*Gammanema* sp. and *Adoncholaimus* sp.), *Gammanema* sp. was temperature tolerant and even increased in relative abundance (Fig. 4D, Table 3). By contrast, the species of trophic group 1B in LDt_0_ (*Rhynchonema* sp. and the two *Theristus* species) disappeared in the high temperature treatment (Fig. 4D, Table 3). Similarly, many of the species representing 1B in the HDt_0_ (*Xyala* sp.1, *Xyalidae gen.1*, *Rhynchonema* and *Omicronema*) were lost in the high temperature treatment, but *Xyala* sp.2 and *Richtersia* sp. survived (Fig. 4D, Table 3). Functional group 2A (epistrate feeders) were represented by 3 and 2 species in HD_36_ and LD_36_ respectively. In both, *Desmodora* sp.1 (HD and LD), *Microlaimus* sp.1 (HD and LD), and *Metachromadora* sp.1 (HD) survived the high temperature exposure (Fig. 4D, Table 3).

## Discussion

In the present study, we assessed the stress response of two intertidal nematode communities, differing in environmental origin and in diversity. Given its environmental background, we expected the low diversity community to cope better with stressful conditions and lose relatively fewer species. On the other hand, according to the IH, we expected that the high diversity community would maintain its functional attributes despite species loss, due to their functional redundancy. In order to test this we exposed natural nematode assemblages of contrasting diversity levels and environmental background to a stressful temperature.

Although the goal of the experiment was to study the effect of high temperature, the experimental enclosure had an effect on abundance and biomass in both HD and LD. Something similar happened in a mesocosm experiment on meiobenthic communities, where abundance decreased significantly between time_0_ and control samples in one of the two experimental communities, whereas in the other, abundance remained constant during the whole experimental duration of 16 weeks [48]. On the other hand, dos Santos [49] was able to maintain abundance and diversity unaltered during 30 days in small (300 ml total, 100 ml sand) sandy beach microcosms. In our study, we set up time_0_ controls and microcosms at a control temperature of 31°C. We were thus able to separate the effects arising from the enclosure and the high temperature, and therefore our hypotheses and predictions were not compromised.

As expected, the LD contained a smaller, but more stress tolerant species pool, whereas the HD was generally more vulnerable to stress, as it lost a significant number of species due to the high temperature. Although our environmental results (grain size, chlorophyll*-a* and organic matter content) imply only a slight difference between the two sampling stations, tidal-related conditions (*e.g.*, exposure to air) were different between the HD and the LD site (Fig. S2). Nematode diversity is related to beach morphodynamics [50], [51] and to physical and chemical gradients [24], [52] among other factors; consequently, the two field assemblages differed significantly in species richness and composition. Given its environmental background, the LD maintained a higher similarity with its original state compared to the HD, which became very different from its original composition. The higher resistance of the LD assemblage, reflected by its non-significant diversity loss, presumably resulted from its exposure to a naturally more stressful environment in the high intertidal where longer exposure and insulation times prevail during low tide. Something similar happened when community stress-response to organic enrichment was tested for nematode assemblages from sandy and muddy intertidal regions [53]. Although the mud-community was less diverse, the effect of organic enrichment had a less drastic effect on species loss, as it was originally better adapted to higher loads of organic matter. In our study, four of the five species contributing to the top 90% discriminating taxa in LD_36_ (Table 3) increased in abundance in HD_36_ (*Perepsilonema* sp., *Desmodora* sp.1, *Ceramonema* sp.3 and *Microlaimus* sp.1), and the difference between the HD community and LD_t0_ decreased slightly but steadily with increasing temperature (HD_t0_-LD_t0_: 77.28, HD_31_-LD_t0_: 74.08, HD_36_-LD_t0_: 70.2). We propose three not mutually exclusive hypothetical mechanisms pertaining to the dominant species surviving the high-temperature stress: 1) increased tolerance: the species exhibit higher tolerance for high temperature and/or other effects of the temperature treatment (*e.g.*, lower oxygen availability), 2) competitive release: nematodes benefit from the lower abundance or disappearance of other species, and 3) increased food supply: a higher bacterial biomass allows rapid population development. Our experiment provides evidence for the first, without excluding the other two. The fact that the LD exhibited higher stress resistance than the HD in terms of diversity changes contradicts the “sampling effect” [54], which states that HD communities are generally more stress resistant because of their higher probability of containing stress-resistant species.

The trophic composition of the two assemblages changed considerably and in different directions, even if the *ITD^−1^* did not reflect it. Although this seems contradictory at first sight, it is not: a high *ITD^−1^* value reflects the dominance of one functional group, whereas a low value reflects an even distribution of all functional groups. However, it gives no indication about which functional groups are present nor about their relative contribution. Therefore, although two assemblages may have similar *ITD^−1^* values, it does not necessarily mean that their trophic composition is similar as well. The HD assemblage lost the entire functional group of predators and omnivores (2B), representing a trophic level that does not overlap with any of the other four trophic guilds. Predatory nematodes are often sensitive to stress, given that they are often long-living species at the top of the meiofaunal food web accumulating irregularities and losses from lower trophic levels, and depending on the biomass of other nematodes [55], [56]. If food availability was the only (or most important) determining factor for the survival of predators, we would expect predators to survive in the HD rather than the LD assemblages. However this was not the case in our study: predators were present in all three LD assemblages (t_0_, 31 and 36) despite the reduced abundance and community biomass, but they were missing in the HD_31_ and HD_36_. In the HD, *Enoploides* and *Epacanthion* disappeared due to the enclosure effect, whereas in the LD, *Gammanema* persisted through the stressful treatment, indicating that this species is not only temperature stress resistant, but also that it is probably an omnivore or scavenger rather than an obligate predator, and that it may be able to switch from one food source to another depending on availability. In the natural environment, the loss of large predatory nematodes may have a considerable impact for the benthic food web in terms of energy transfer from meio- to macrofauna and higher trophic levels, since larger nematodes are more prone to predation by hyperbenthos [7]. On the other hand, the functional implications of the loss of trophic group 1B (unselective deposit feeders) in the LD is less evident. Species belonging to the trophic group 1B are not particularly big on average. Moreover, their ecological function could overlap, at least partly, with nematodes from functional groups 1A and 2A, since they have also been observed feeding on bacteria and microalgae [57]. The general increase of the feeding guild 1A (bacteria feeders) in the high temperature treatments may be due to the potential increase in bacterial biomass. Such an increase in bacteria feeding species also occurred during experimental enrichment of organic matter, which, as a secondary effect, caused increased bacterial growth [53].

The results of this study do not support the IH, implying that functional redundancy in the observed intertidal marine nematode assemblages is low as per our measured response variables. However, it is important to specify that the trophic function is just one of multiple functions nematode communities are involved in, and redundancy patterns may vary among multiple functions. Our expectation in the case of functional redundancy would have been that the HD community would maintain its functionality better than the LD community, because the latter does not have a similar buffer against species loss. However, despite a more prominent loss of species in the HD, both LD and HD were similarly impaired in their functionality. Against this background, we find some evidence for different models relating diversity and ecosystem functioning, such as the Rivets Model, which predicts that: a) a community of high diversity functions better than one of low diversity and b) the loss of functionality is contingent on the loss of species. These predictions are at least partly met with our data in both the HD and the LD assemblages. Both exhibited a better functioning (*i.e.* higher abundance, higher community biomass) with higher species richness and diversity, and both showed a similar overall pattern of loss of functionality following a loss of taxonomic richness. We also find some support for the Idiosyncrasy Model, which states that the functional response depends on the species lost and therefore cannot be predicted. In the present study, the stressful environment caused the HD assemblage to lose its entire trophic group of predators (2B), which consisted of *Enoploides* and *Epacanthion*. The LD assemblage, on the other hand, maintained its trophic group 2B, represented by *Gammanema* after the enclosure and the temperature effect, but lost its trophic group of detritus feeders due to the high temperature effect. Thus, depending on the species lost, the trophic composition of the assemblage changed in different directions, but this change could not have been predicted *a priori*. On the other hand, there is also some evidence against the Idiosyncrasy Model since the impairment of the functionality in terms of biomass did not depend on species identity. Different species became relatively more abundant in the HD than in the LD. Moreover, the community dissimilarity between HD_t0_ and HD_36_ was much higher (70. 08) than between LD_t0_ and LD_36_ (52.04). Yet, the loss of functionality occurred at similar levels in both HD and LD. This stands in contrast to the Idiosyncrasy Model since the impairment of the functionality did not depend on the identity of the species lost. Consequently, based on our data we cannot side with any of the three models (IH, Rivets or Idiosyncrasy) conclusively, but we clearly do not find evidence for species redundancy. This concurs with other studies on selected bacterivore nematode species, where only incomplete functional overlap could be detected between even closely related species, and species identity rather than species richness affected functionality [4], [20]. Furthermore, it concurs with other studies on benthic macrofauna, where species identity, but not richness had a significant effect on ecological processes such as nutrient generation [15] or oxygen consumption [16]. Our results, in combination with those from other studies on benthic communities reveal that the relationship between species diversity and the functioning of the system is not a linear but a more complex one. Although other studies on other systems have shown clear redundancy effects of high diversity communities, we cannot presently affirm that functional redundancy exists in nematode assemblages at the diversity levels and the functions we measured. This is all the more surprising, since nematodes are one of the most diverse phyla of metazoan organisms [58] and it leaves the question open, whether functional redundancy occurs at all in metazoans.

In the present study, we set out to test, for the first time, the structural and functional changes of a natural subtropical meiofaunal community in response to increased water temperature. Our results successfully showed that changes in nematode communities are not consistent with the prevalence of functional redundancy, and hence are of consequence to hypotheses relating diversity with ecosystem function. Future experiments on intertidal nematode communities addressing this issue will benefit from the following recommendations: 1) more functional variables (in addition to trophic functions) could be investigated, as nematodes are involved in many different functions in the intertidal, with potentially varying patterns of redundancy. This will greatly improve our understanding in the current diversity-redundancy debate. 2) The experimental setup could incorporate a more realistic simulation of the intertidal environment such as tidal action, as constant water cover may act as an additional stress source on the communities. 3) Additional efforts could be made to better tease apart the effects of community composition and diversity levels in order to identify their potentially independent effects on ecosystem functioning. Admittedly, this represents a major challenge given the difficulty of finding species assemblages from the same type of environment in which diversity and taxonomic composition are not at least partially confounded. 4) Quantitative replicate sampling during the course of the experiment will provide evidence of the temporal changes and response of the community to the environmental stressor and allow testing additional hypotheses regarding the onset and rate of structural and functional changes of the community. 5) Finally, additional environmental variables such as algal and bacterial growth could be measured in the microcosms. This will help clarify causal relationships between the proxies of community function and environmental changes. For example, an increase in a bacteria-feeding guild could be linked to an increase in bacterial growth.

## Supporting Information

Figure S1 **Beach topographic profile of the sampling location.** Sampling sites are indicated with red arrows. Sampling station E6 is the high diversity site, E8 is the low diversity site.
(TIF)

Figure S2 **Difference in exposure time between the high diversity and the low diversity site (blue line) for the month of sampling.** The red line indicates the monthly mean (3.84 h). Blue and grey shaded areas indicate spring and neap tides respectively.
(TIF)
