# Supplementary figures and images for: Assessing the Response of Nematode Communities to Climate Change-Driven Warming: A Microcosm Experiment
Source: PLoS One. 2013 Jun 18;8(6):e66653. doi: 10.1371/journal.pone.0066653 (PMC3688992; doi:10.1371/journal.pone.0066653)

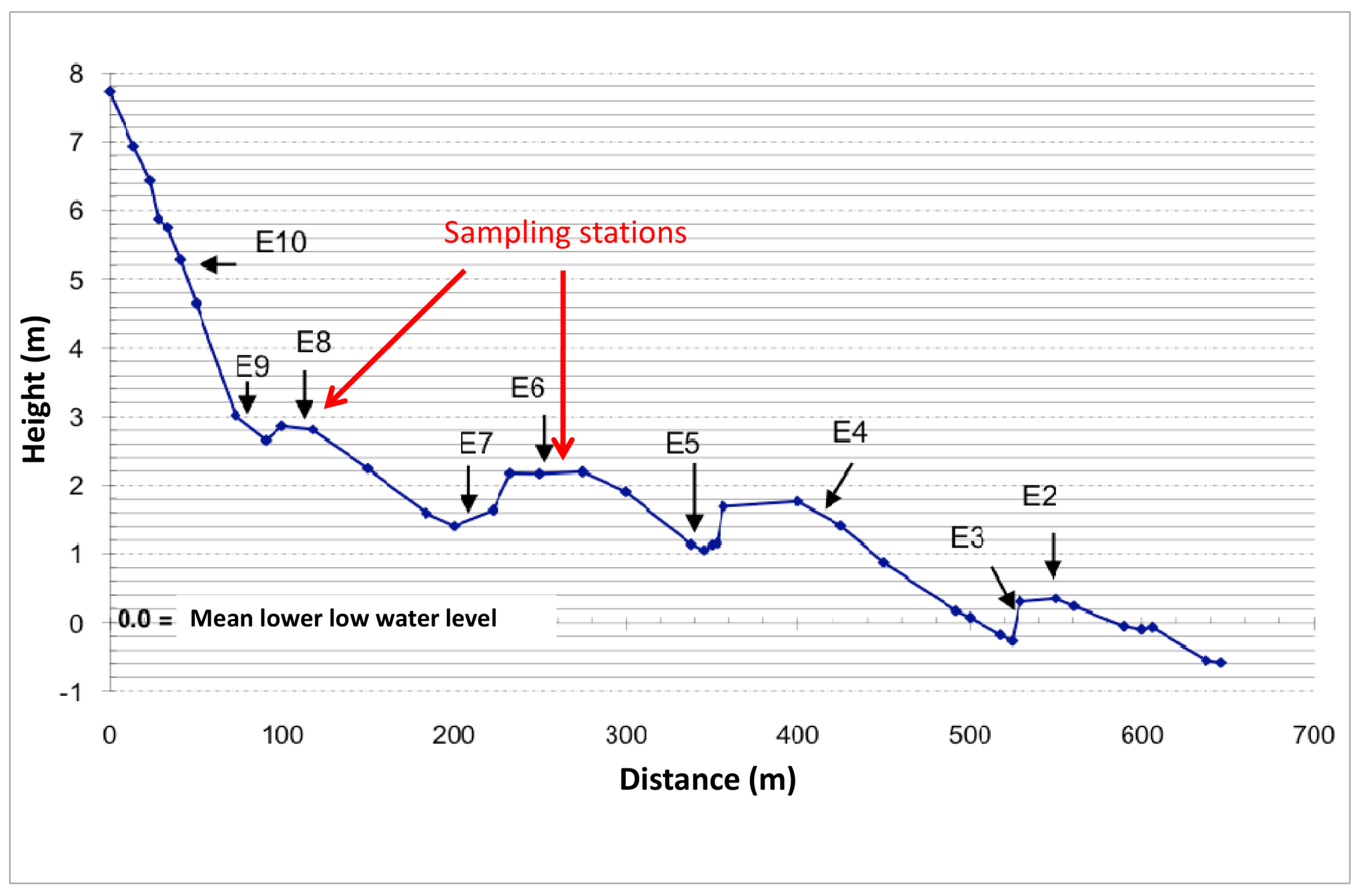

Supplement: Figure S1 — Beach topographic profile of the sampling location. Sampling sites are indicated with red arrows. Sampling station E6 is the high diversity site, E8 is the low diversity site. (TIF) [file pone.0066653.s001.tif]

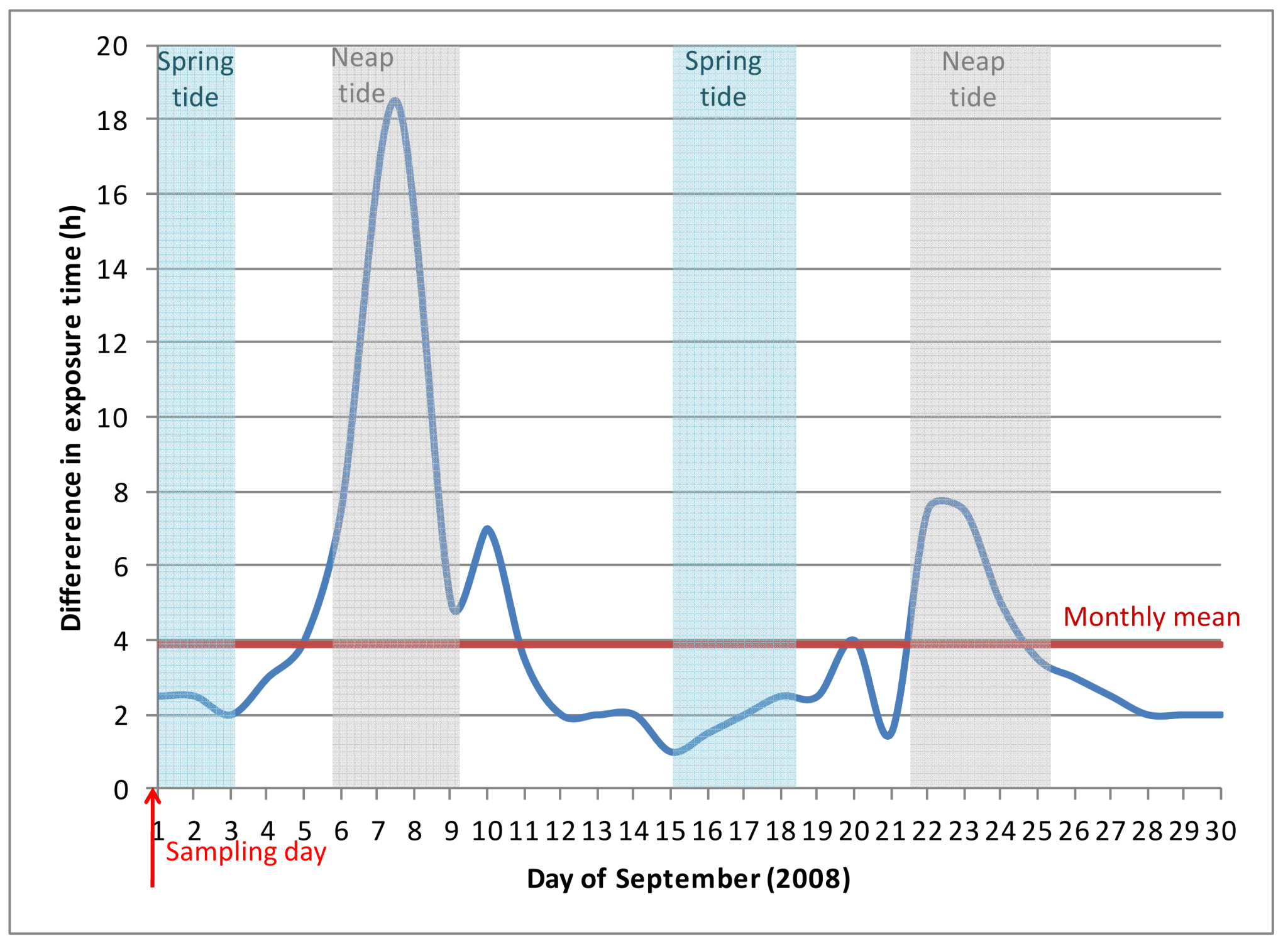

Supplement: Figure S2 — Difference in exposure time between the high diversity and the low diversity site (blue line) for the month of sampling. The red line indicates the monthly mean (3.84 h). Blue and grey shaded areas indicate spring and neap tides respectively. (TIF) [file pone.0066653.s002.tif]
